# Coexistence of myocardial infarction, spontaneous coronary artery dissection, and Takotsubo syndrome: a case evaluated by intravascular ultrasound and cardiac magnetic resonance imaging

**DOI:** 10.1093/ehjcr/ytaf084

**Published:** 2025-02-18

**Authors:** Yosuke Uchida, Masataka Suzuki, Hiromi Hashimura, Hiroshi Eizawa

**Affiliations:** Department of Cardiology, Kobe City Nishi-Kobe Medical Center, 5-7-1, Kojidai, Nishi-ku, Kobe, Hyogo 651-2273, Japan; Department of Cardiology, Kobe City Nishi-Kobe Medical Center, 5-7-1, Kojidai, Nishi-ku, Kobe, Hyogo 651-2273, Japan; Department of Radiology, Kobe University Graduate School of Medicine, Kobe, Japan; Department of Cardiology, Kobe City Nishi-Kobe Medical Center, 5-7-1, Kojidai, Nishi-ku, Kobe, Hyogo 651-2273, Japan

A 71-year-old female with a history of dyslipidaemia was referred to our institution due to chest pain. The patient experienced no preceding emotional or physical stress. Electrocardiography revealed ST-segment elevation in leads V3–V6. Emergency coronary angiography demonstrated occlusion in the distal left anterior descending artery (LAD). Coronary reperfusion with thrombolysis in myocardial infarction (MI) 3 flow was achieved via guidewire crossing. Intravascular ultrasonography (IVUS) revealed a false lumen with intramural haematoma at the occlusion site, consistent with spontaneous coronary artery dissection (SCAD) (*Figure 1A–C*, Supplementary material online, *Videos S1–S3*). Transthoracic echocardiography showed mid-to-apical hypokinesis with basal hyperkinesis, suggesting another condition aside from MI in the distal LAD (see Supplementary material online, *Videos S1*–*S3*). Late gadolinium enhancement (LGE) of cardiac magnetic resonance imaging (CMR) exhibited significant high-signal intensity in the apex and mildly high-signal intensity in the transmural layers of the mid-ventricle. T1 and T2 values were gradually increased from the base to the apex (*Figure 1D–F*). These findings indicated the coexistence of apical MI due to SCAD and Takotsubo syndrome (TTS). The chest pain improved following coronary reperfusion; however, chest discomfort persisted for several days. Medical therapy, including aspirin and enalapril, was administered; a beta-blocker was pending because of an intermittent 2:1 atrioventricular block. Left ventricular wall motion abnormality was normalized except for the infarcted apex, and left ventricular ejection fraction improved from 50% at admission to 60% at discharge.

**Figure 1:**
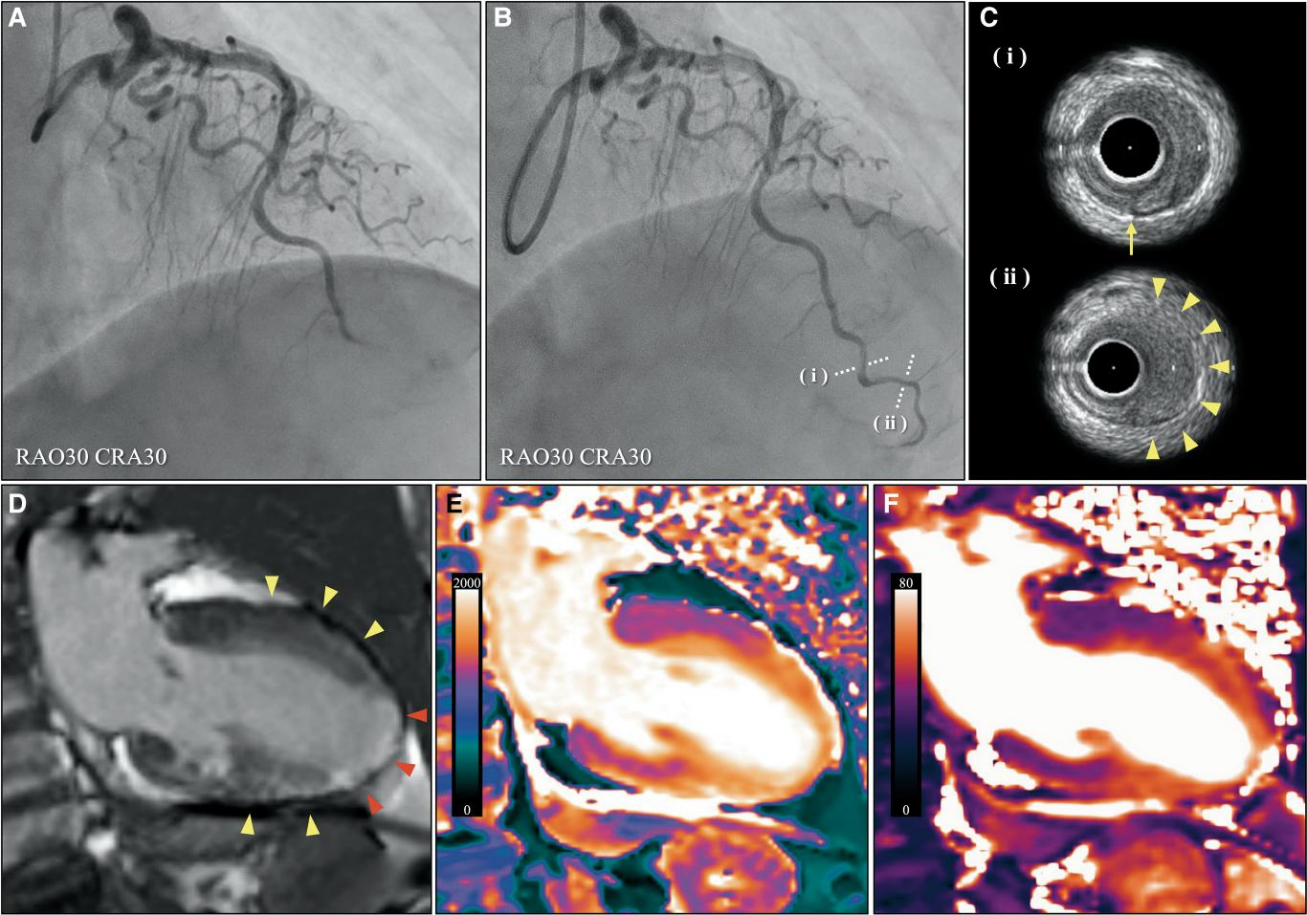
(*A*) Initial and (*B*) post-percutaneous coronary intervention coronary angiography reveals occlusion in the distal left anterior descending artery, which is recanalized through guidewire crossing. (*C*) Intravascular ultrasonography shows an intima tear (arrow) and an intramural haematoma (arrowheads) in each lesion (i, ii) visible on *B*, suggesting spontaneous coronary artery dissection. (*D*) Late gadolinium enhancement of cardiac magnetic resonance imaging demonstrates significant high-signal intensity in the infarcted apex (arrowheads) and mildly high-signal intensity in the transmural layers of the non-infarcted mid-ventricle (arrowheads). (*E*) T1 and (*F*) T2 mapping demonstrate gradually increasing T1 and T2 values from the base to the apex [1227 ms at the base, 1341 ms at the mid, and 1658 ms at the apex in T1 mapping (normal, 1182 ± 24 ms); 40 ms at the base, 51 ms at the mid, and 61 ms at the apex in T2 mapping (normal, 41 ± 1 ms)].

Both SCAD and TTS predominantly affect females and can be triggered by emotional or physical stress. Recent reports have documented the coexistence of SCAD and TTS. Since SCAD of the LAD typically presents as apical hypokinesis, which is also observed in TTS, the coexistence of both conditions could be underdiagnosed.^1^ On CMR, LGE shows absent or mild enhancement in TTS compared to that in MI.^2^ T1 and T2 mapping have also been utilized to assess myocardial tissue characterization of TTS.^3^ In this case, mildly enhanced LGE and slightly increased T1 and T2 values in the non-infarcted mid-ventricle indicated interstitial expansion from inflammation and transient fibrosis caused by TTS. Meanwhile, dense LGE and highly increased T1 and T2 values in the apex indicated necrosis caused by MI. This is the first report presenting the coexistence of MI, SCAD, and TTS, evaluated using IVUS and CMR. CMR is useful for evaluating myocardial tissue differentiation of MI and TTS and their coexistence.

## Supplementary Material

ytaf084_Supplementary_Data

## Data Availability

The data underlying this article will be shared upon reasonable request to the corresponding author.

## Supplementary material

**Consent:** Written informed consent for submission and publication of this case report including images has been obtained from the patient in line with COPE guidance.

**Funding:** None declared.
